# Viral DNA in submandibular gland tissue with an inflammatory disorder

**DOI:** 10.1080/20002297.2024.2345941

**Published:** 2024-04-26

**Authors:** Noora Keski-Säntti, Elin Waltimo, Antti Mäkitie, Jaana Hagström, Maria Söderlund-Venermo, Timo Atula, Caj Haglund, Saku T. Sinkkonen, Maria Jauhiainen

**Affiliations:** a Department of Virology, University of Helsinki, Helsinki, Finland; b Otorhinolaryngology – Head and Neck Surgery, University of Helsinki and Helsinki University Hospital, Helsinki, Finland; c The Doctoral Programme in Clinical Research, Faculty of Medicine, University of Helsinki, Helsinki, Finland; d Department of Oral and Maxillofacial Diseases, University of Helsinki, Helsinki, Finland; e Research Program in Systems Oncology, Faculty of Medicine, University of Helsinki, Helsinki, Finland; f Department of Pathology, University of Helsinki and Helsinki University Hospital, Helsinki, Finland; g Department of Oral Pathology and radiology, University of Turku, Turku, Finland; h Research Programs Unit, Translational Cancer Medicine, University of Helsinki, Helsinki, Finland; i Department of Surgery, University of Helsinki and Helsinki University Hospital, Helsinki, Finland

**Keywords:** Chronic sialadenitis, DNA viruses, immunoglobulin G4 related disease, Sialolithiasis, Polymerase chain reaction

## Abstract

**Background:**

The etiology behind different types of chronic sialadenitis (CS), some of which exhibit IgG4 overexpression, is unknown. Further, IgG4-related disease (IgG4-RD) commonly affects the submandibular gland, but its relationship to IgG4-overexpressing CS, and the antigen triggering IgG4 overexpression, remain unknown.

**Materials and Methods:**

By qPCR, we assessed the presence of 21 DNA-viruses causing IgG4 overexpression in submandibular gland tissue from patients with IgG4-positive and IgG4-negative CS. Healthy submandibular glands and glands with sialolithiasis without CS were used as controls. We examined the distribution of HHV-7, HHV-6B and B19V DNA, within virus PCR-positive tissues with RNAscope in-situ hybridization (RISH).

**Results:**

We detected DNA from seven viruses in 48/61 samples. EBV DNA was more prevalent within the IgG4-positive samples (6/29; 21%) than the IgG4-negative ones (1/19; 5.3%). B19V DNA was more prevalent within the IgG4-negative samples (5/19; 26%) than the IgG4-positive ones (4/29; 14%). The differences in virus prevalence were not statistically significant. Of the IgG4-RD samples (*n* = 3) one contained HHV-6B DNA. RISH only showed signals of HHV-7.

**Conclusions:**

None of the studied viruses are implicated as triggering IgG4-overexpression in CS. Although our results do not confirm viral etiology in the examined conditions, they provide valuable information on the prevalence of viruses in both diseased and healthy submandibular gland tissue.

## Introduction

Chronic sialadenitis (CS) is usually of obstructive origin, induced by sialolithiasis or strictures caused by acute microbial infections or iatrogenic factors [1–3]. Autoimmune disorders, specifically Sjögren’s syndrome, may also underlie CS [1,2]. Chronic sclerosing sialadenitis (CSS, ‘Küttner’s tumor’) represents a specific form of CS, with distinct clinical and histological features [4]. CSS typically affects the submandibular glands and presents as a firm, tumor-like mass. Histologically CSS is characterized by lymphoplasmacytic infiltrates, atrophy and dense fibrosis [3]. In the recent decades, CSS has been considered a manifestation of immunoglobulin G4-related disease (IgG4-RD) [4,5], a systemic fibroinflammatory disorder presenting with tumor-like lesions rich in IgG4-positive plasma cell infiltrates, and elevated serum IgG4 levels [6,7]. Recent studies however indicate that albeit IgG4-positive plasma cell infiltrates are often found in CSS, it is exceedingly rarely related to genuine IgG4-RD [8–10]. Further, inflammatory infiltrates presenting high numbers of IgG4-positive plasma cells can be found in other, non-specific forms of CS as well [10–12]. These conflicting findings have led to a hypothesis of a specific form of submandibular sialadenitis, separate from true IgG4-RD, where chronic inflammatory infiltrates produce overexpression of IgG4-positive plasma cells in the affected salivary gland. However, the trigger behind the overexpression of IgG4 in some forms of sialadenitis is unknown [9,10,12].

Infectious agents play an important role as etiological factors in human disease, such as cancer [13] and autoimmune disorders [14]. In addition to their primary infections, the role of DNA viruses as causative agents in human disease development is not yet fully understood. The salivary glands are highly exposed to microbes due to their anatomical connection to the oromucosal pathway, and therefore present a compelling target to study virus etiology. However, large cohort studies with a systematic screening strategy to investigate the viral involvement in CS have not been performed. Furthermore, the role of viruses in the development of CS, or the IgG4 overexpression seen in some types of sialadenitis, is unknown.

Viruses in the *Orthoherpesviridae* family are highly prevalent among healthy individuals and have the ability of life-long latent infection and reactivation. Association of herpesviruses to chronic inflammatory disease and cancer development has been reported; but for part of the viruses in the *Orthoherpesviridae* family, knowledge of their ability to cause disease is still deficient [15–17]. Human herpes virus (HHV) −6B and −7 persist in the salivary glands [18,19] and are secreted in saliva [20]. The association between IgG4-positive sialadenitis and Epstein-Barr virus (EBV) has been investigated in a few small cohorts [21,22], but evidence supporting this is still deficient. Human parvovirus B19 (B19V) has a high seroprevalence (40–80%) among adults, and has been reported to persist in multiple tissues, including the tonsils [23,24]. B19V capsid protein VP1 has been shown to induce delayed IgG4 responses [25], and its role in inflammatory and autoimmune diseases has been investigated, but no evident association has been found [13,26,27]. Other members of the *Parvoviridae* family: the cuta-, bufa-, and tusaviruses (CuV, BuV, and TuV), and human bocaviruses 1–4 (HBoV1–4); and *Polyomaviridae* family: the Merkel cell polyomavirus (MCPyV), JC polyomavirus (JCV), BK polyomavirus (BKV), and trichodysplasia spinulosa virus (TSPyV) have only been discovered within the recent decade. Little is known about these viruses as causative agents in human disease, moreover their presence in salivary gland tissues has not been previously studied [13,28–31].

Hence, our aim was to investigate the presence of 21 DNA viruses in the submandibular glands of patients with CS. We used a systematic screening strategy for DNA viruses of the *Orthoherpesviridae*, *Parvoviridae*, and *Polyomaviridae* families. We wanted to assess if differences in virus prevalence between non-specific and more specific forms of sialadenitis, such as CSS, could be detected or whether the salivary glands harbored the same viruses. Both IgG4-positive and –negative samples were included to determine a possible association of a viral triggering antigen behind the overexpression of IgG4 in some forms of salivary gland infections, and further examine a possible involvement of viral antigens in true IgG4-RD.

## Materials and methods

### Ethics

The research ethics committee at the Helsinki University Hospital (record number 192/13/03/02/16) approved the study design, and an updated institutional research permission was granted (original permit number, §41/2017; updated permit number §45/2022). The tissue samples used in this study were retrospectively collected over a long time period, between the years 2000–2017. For the collection, management, and analysis of these samples we had a permit from the National Supervisory Authority of Welfare and Health (record number 004/06.01.03.01/2012), and the Finnish Medicines Agency (record number 2021/006901). The management of samples obtained after the year 2013 was done in accordance with the Biobank act (688/2012).

### Patient data and clinical specimens

The patients in this study were selected from cohorts formed in two earlier retrospective studies, which examined the association of IgG4-RD and IgG4-positive plasma cell infiltrates in a cohort of patients diagnosed with CSS and a cohort of patients diagnosed with CS and sialolithiasis [10,12]. From this patient series we formed two study groups: an IgG4-positive group and an IgG4-negative group. IgG4-positivity in a tissue sample was defined as a finding of ≥ 70 IgG4-positive plasma cells in a high-power field of x40 magnification [10,12]. In the IgG4-positive group, we included all the samples that were confirmed as IgG4 positive or as true IgG4-RD in the two previous studies. In the IgG4-negative group, we included samples without IgG4 overexpression. The samples in both the IgG4-positive group and the IgG4-negative group were further divided into subgroups based on their histologically assigned diagnoses: CSS, nonspecific CS, and/or sialolithiasis and sialolithiasis without associated CS. As controls, we acquired submandibular gland samples from healthy salivary glands removed during neck dissection. The samples from patients with IgG4-negative sialolithiasis without associated CS were additionally comparative to healthy tissue, as these samples featured none or only locally situated nonspecific, very mild, inflammatory infiltrates. The tissue samples were acquired from Helsinki Biobank and clinical patient data including sex, age at the time of surgery, and the preoperative diagnosis were collected from the Helsinki and Uusimaa Hospital district patient records and pathology reports.

Tissue was harvested from formalin-fixed paraffin-embedded (FFPE) tissue blocks as 2 mm punch biopsies in a PCR-sterile manner. Punch biopsies were stored in 1.5 ml microcentrifuge tubes until the DNA extraction.

### DNA extraction and virus detection

Paraffin was dissolved from the tissue samples by immersing the samples in xylene twice at room temperature for 30 minutes. DNA was extracted using the QIAamp DNA mini kit (Qiagen, Heiden, Germany) according to the manufacturer’s protocol with some modifications. A volume of 40 μl of proteinase K was used to dissolve tissue and the extracted DNA was eluted in 100 μl AE buffer. DNA extracts were stored at −20°C until the viral assays.

The cell count was quantified by the amplification of the human *RNase P* gene in all samples, as described [32]. A three-tube multiplex qPCR assay [33] was applied to target each virus in the *Orthoherpesviridae* group (herpes simplex virus type 1 and 2 (HSV-1, HSV-2), and varicella-zoster virus (VZV); HHV-6A, HHV-6B, and HHV-7; EBV, cytomegalovirus (CMV), and HHV-8). For the members of *Parvoviridae*, B19V was detected and quantified with a pan-B19 qPCR, targeting the NS1 genes of all 3 genotypes [32] and HBoV1–4 in turn by a multiplex qPCR, targeting the NS1 regions of all 4 bocaviruses [34]. CuV, BuV, and TuV were amplified and quantified by using a multiplex qPCR [29,35] targeting the NS1 region of BuV and VP2 regions of TuV and CuV. For the members of the *Polyomaviridae* group (MCPyV, JCV, BKV, TSPyV), a singleplex qPCR was performed separately for each virus targeting the VP1 region, as described [36,37].

Each qPCR reaction consisted of Maxima probe qPCR Master Mix (Thermo Scientific) with or without ROX as passive reference dye or 2×Taqpath Proamp Multiplex Master Mix (Fisher), the (appropriate amount of) gene-specific forward and reverse primers and probe, template (2.5–5 μl), and molecular biology-grade H_2_O to a final volume of 20–25 μl. After an initial denaturation step of 10 min at 95°C, 40–45 cycles were performed at 95°C for 15 s and at 60–62°C for 60 s. Molecular biology grade water was included in every PCR reaction to serve as a negative control. Each viral target amplicon containing ten-fold diluted plasmids (10^1^–10^6^) was used for a standard curve and positive controls. All real-time qPCR assays were performed with AriaMx Real-Time PCR System (Agilent Technologies, Santa Clara, CA). The PCR runs of the virus-positive samples were made as duplicates.

The master mix components, samples, and plasmids were handled in laminar hoods in separate rooms. Filtered tips and single-use disposable materials were used in each step. For the viruses detected by qPCR, a positive result from duplicate wells, or if confirmed by sequencing, was defined as positive. Viral DNA load was compared to *RNase p* values to determine the viral DNA copy number per one million cells (cpm).

### RNAscope in-situ hybridization

RNAscope in-situ hybridization (RISH) was performed on three tissue samples to evaluate the presence and distribution of viruses in the tissue and to assess whether the viruses were concentrated in the inflamed or the surrounding tissue. Tissue samples with high viral loads (>1000 cpm) were selected. This criterion was met for samples with B19V, HHV-6B, and −7 DNA. A sample for B19V was from a patient with IgG4-negative sialolithiasis with CS, for HHV-6B from a patient with IgG4-positive sialolithiasis with CS, and for HHV-7 with IgG4-positive CSS.

RISH was performed on FFPE tissue sections using the RNAscope 2.0 HD Red Chromogenic Reagent Kit (Advanced Cell Diagnostic, CA) according to the manufacturer’s instructions. First, FFPE samples were cut into 5-μm tissue sections and mounted onto Superfrost microscope slides. Tissue sections were then deparaffinized twice in xylene, followed by dehydration in ethanol. Next, the sections were incubated 10 min in hydrogen peroxidase after which target retrieval was performed at 95°C for 15 min. Slides were then rinsed with distilled water and left to dry at room temperature overnight. Tissue sections were then treated with 10 μg/mL protease (Sigma-Aldrich, St. Louis, MO) at 40°C for 30 min in a HybEZ hybridization oven (Advanced Cell Diagnostics, Hayward, CA).

To target the DNA or RNA for each virus, probes targeting the B19V *NS*, HHV-7 *U4*, and HHV-6B *U38* genes were hybridized to the viral nucleic acid in the samples selected for the assay. To assess the method, a probe targeting the human *PPIB* gene served as a technical positive control and a probe targeting the bacterial *dapB* gene as a technical negative control. Furthermore, a virus PCR-negative tissue sample was included in each assay as a negative control to assess specificity and any possible background noise. After the hybridization of the probes, the signal was amplified by a multi-step process following the manufacturer’s instructions. For signal detection, the kit FastRed dye (RED-A and RED-B) was added on each sample, after which the sections were counterstained with hematoxylin. After dehydration at 60°C for one hour, the samples were mounted with EcoMount and left to dry for 2 days, after which they were scanned at x40 magnification. Target DNA or RNA was identified as red chromogenic dots.

### Statistics

The statistical analyses were performed using RStudio software (version 2022.12.0 + 353), and SPSS (version 28.0.). Fisher’s exact test was applied to calculate the differences in the virus prevalence and Mann Whitney U -test to determine differences in the viral DNA cpm between the IgG4-negative and -positive samples, and the samples representing healthy tissue. A *p*-value <0.05 was considered statistically significant.

## Results

### Patient characteristics

A total of 61 patients were included in the study. These included the IgG4-positive (*n* = 29/61; 48%) and IgG4-negative samples (19/61; 31%), and the control samples representing healthy salivary gland tissue (13/61;21%). The patient groups are presented in Table 1. Sex and age were assessed from each group, excluding the three control samples acquired from neck dissection, for which clinical data were not accessible. The distribution of patients between the different sub-groups is presented in Table 1. In the IgG4-positive and IgG4-negative groups, respectively, 15 (52%) and 8 (42%) patients were male. The mean ages at the time of surgery was 53 years (range 25–86) in the IgG4-positive group and 56 years (range 35–79) in the IgG4-negative group. Of the patients in the control group, for whom clinical data was available, 6 (60%) were male, and had a mean age of 44 years (range 19–61) at the time of surgery.

**Table 1.** Results of virus findings in the IgG4-positive group, the IgG4-negative group, and the control group comprising of samples representing healthy salivary gland tissue.

		*Orthoherpesviridae*				*Parvoviridae*		*Polyomaviridae*
	Virus-positive samples (%)*	HHV-6B	HHV-7	EBV	VZV	B19	HBoV	MCV
IgG4-positive samples, all* (n = 29)	**21 (72.4)**	**15 (51.7)**	**13 (44.8)**	**6 (20.7)**	**1 (3.5)**	**4 (13.8)**	**1 (3.5)**	**0**
Nonspecific sialadenitis (*n* = 5)	2	2	2	1	0	2	0	0
Chronic sclerosing sialadenitis (*n* = 8)	7	4	4	2	1	2	0	0
Sialolithiasis with sialadenitis (*n* = 11)	10	7	7	3	0	0	1	0
Sialolithiasis without sialadenitis (*n* = 2)	1	1	0	0	0	0	0	0
IgG4 related disease (*n* = 3)	1	1	0	0	0	0	0	0
IgG4-negative samples, all* (n = 19)	**15 (78.9)**	**9 (47.3)**	**10 (52.6)**	**1 (5.3)**	**0**	**5 (26.3)**	**0**	**1 (5.3)**
Nonspecific sialadenitis (*n* = 10)	9	6	8	1	0	1	0	0
Chronic sclerosing sialadenitis (*n* = 9)	6	3	2	0	0	4	0	1
Healthy salivary gland tissue, all* (n = 13)	**12 (92.3)**	**9 (69.2)**	**11 (84.6)**	**0**	**0**	**3 (23.1)**	**0**	**0**
Sialolithiasis withouth sialadenitis (*n* = 10)	9	6	8	0	0	3	0	0
Healthy glands from neck dissection (*n* = 3)	3	3	3	0	0	0	0	0

*Due to the low patient numbers, the prevalence percentages are given only to ‘all’, where n = 29 in the IgG4-positive group, n = 19 in the IgG4-negative group, and n = 13 in the group representing healthy salivary gland tissue.

#### Presence of virus DNA in salivary gland tissue samples

Altogether 61 samples were analyzed. The *RNase p* values ranged between 0.04–8700 (mean 2300) cells/uL DNA extract. A total of 21 viruses were analyzed. Virus findings are summarized and presented separately for each disease entity in Table 1. Viral DNA from seven viruses, including HHV-6B and −7, EBV, B19V, HBoV, VZV, and MCPyV, was detected in altogether 48/61 (79%) samples. Multiple viral DNAs were found in 28/61 (46%) samples. The maximum number of different viruses detected in a sample was four, Table 2. summarizes the cases with multiple virus findings. The one sample positive for HBoV was negative for all other studied viruses. The remaining 13/61 (21%) samples were negative for all viruses. HSV-1, HSV-2, HHV-6A, CMV, HHV-8, CuV, BuV, TuV, JCV, BKV, and TSPyV were not detected in any of the studied samples. The number of copies of the genomes detected in the tissue by the different qPCRs ranged from 3.2 × 10^1^–3.8 × 10^6^ cpm for HHV6B, 1.1 × 10^2^–4.2 × 10^8^ cpm for HHV7, 1.0 × 10^2^–2.9 × 10^3^ cpm for EBV and 0.6 × 10^1^–5.6 × 10^3^ cpm for B19V. For the viruses detected in only one sample the copy number was 5.9 × 10^1^ cpm for HBoV, 4.2 × 10^2^ cpm for MCPyV, and 2.8 × 10^2^ cpm for VZV.

**Table 2.** Virus co-findings in the IgG4-positive group, the IgG4-negative group, and the control group comprising of samples representing healthy salivary gland tissue.

			2 viruses					3 viruses		4 viruses	
	Samples positive for ≥ 2 viruses (%)*	Mean virus number/sample	*HHV-6B, HHV-7*	*HHV-6B, EBV*	*HHV-6B, VZV*	*HHV-7, EBV*	*HHV-7, B19V*	*HHV-6B, HHV-7, EBV*	*HHV-6B, HHV-7, B19V*	*HHV-6B, HHV-7, EBV, B19V*	*HHV-6B, HHV-7, B19V, MCpyV*
IgG4-positive samples, all* (n = 29)	**11 (37.9)**	**1.4**	**3**	**1**	**1**	**0**	**0**	**3**	**1**	**2**	**0**
Nonspecific sialadenitis (*n* = 5)	2	1.4	0	0	0	0	0	0	1	1	0
Chronic sclerosing sialadenitis (*n* = 8)	3	1.6	0	0	1	0	0	1	0	1	0
Sialolithiasis with sialadenitis (*n* = 11)	6	1.6	3	1	0	0	0	2	0	0	0
Sialolithiasis without sialadenitis (*n* = 2)	0	0.5	0	0	0	0	0	0	0	0	0
IgG4 related disease (*n* = 3)	0	0.3	0	0	0	0	0	0	0	0	0
IgG4-negative samples, all* (n = 19)	**8 (42.1)**	**1.4**	**4**	**0**	**0**	**2**	**0**	**0**	**1**	**0**	**1**
Nonspecific sialadenitis (*n* = 10)	6	1.6	4	0	0	1	0	0	1	0	0
Chronic sclerosing sialadenitis (*n* = 9)	2	1.1	0	0	0	1	0	0	0	0	1
Healthy salivary gland tissue, all* (n = 13)	**9 (69.2)**	**1.8**	**6**	**0**	**0**	**0**	**1**	**0**	**2**	**0**	**0**
Sialolithiasis without sialadenitis (*n* = 10)	6	1.7	3	0	0	0	1	0	2	0	0
Healthy glands from neck dissection (*n* = 3)	3	2.0	3	0	0	0	0	0	0	0	0

*Due to the low patient numbers, the prevalence percentages are given only to ‘all’, where n = 29 in the IgG4-positive group, n = 19 in the IgG4-negative group, and n = 13 in the group representing healthy salivary gland tissue.

Virus DNA was detected in 21/29 (72%) of the IgG4-positive, 15/19 (79%) of the IgG4-negative samples, and in 12/13 (92%) of the samples representing healthy salivary gland tissue. HHV-6B and HHV-7 were the most frequently detected viruses in both study groups and in the control group. DNA from HHV-6B, HHV7 or both was found in 19/29 (66%) of the IgG4-positive samples, in 13/19 (68%) of the IgG4-negative samples and in 12/13 (92%) of the samples representing healthy salivary gland tissue (Table 1). The control group had the highest prevalence of HHV-7 DNA (11/13; 85%), this was significantly higher than the HHV-7 prevalence observed in the IgG4-positive group (13/29; 45%), *p* = 0.021. Furthermore, the copy numbers of HHV-7 were higher in the samples representing healthy tissue (mean viral load 4.0 × 10^7^ cpm) compared to the IgG4-positive (mean viral load 1.8 × 10^4^ cpm), and the IgG4-negative group (mean viral load 2.0 × 10^3^ cpm), *p* = 0.007 and *p* = 0.013 respectively. No statistically significant differences in the HHV-6B and HHV-7 prevalence or mean DNA copy numbers between the IgG4-negative and -positive groups were found. EBV DNA was found more frequently in the IgG4-positive samples (6/29; 21%) compared to the IgG4-negative samples (1/19; 5%), *p* = 0.219. B19V DNA prevalence was higher among the IgG4-negative samples (5/19, 26%) than the IgG4-positive samples (4/32, 14%). Also, the mean B19V DNA copy number seemed higher in the IgG4-negative samples compared to the IgG4-positive samples (2514 cpm and 165 cpm, respectively), but the difference remained insignificant (*p* = 0.188). B19V DNA was found in 3/13 (23%) samples, which represented healthy tissue. VZV was found in one patient with IgG4-positive CSS, HBoV in one patient with IgG4-positive sialolithiasis with CS, and MCPyV in one patient with IgG4- negative CSS.

The true IgG4-RD group included three patients only. Viral DNA of HHV-6B was found in only one of these samples, whereas in the two others, no viruses were detected (Table 1).

### RNAscope in-situ hybridization

**Figure 1.**
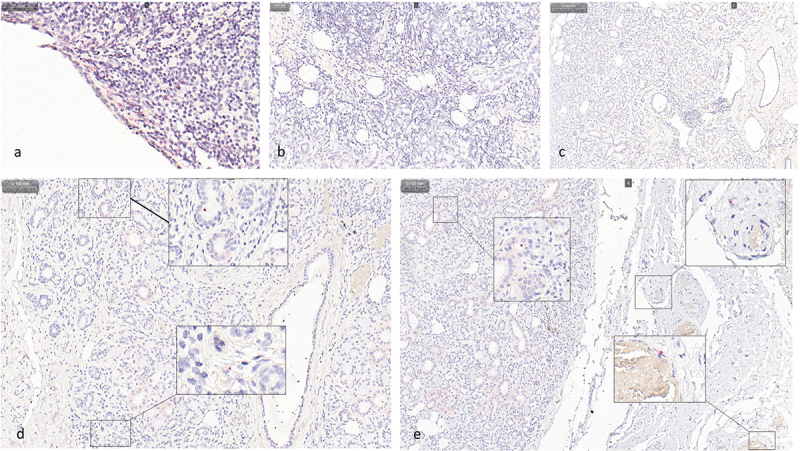
RNAscope ™ 2.0 HD Red on salivary gland FFPE samples. a, RISH with the technical positive *PPIB* probe control. b, RISH with the technical negative *DapB* probe control. c–e, RISH with the HHV-7 *U4* probe on a HHV-7 qPCR-negative sample (C) and HHV-7 qPCR-positive sample from a patient with IgG4-positive CSS (D, E). Positive signals are presented as red punctuated dots. Original magnification × 40. Abbreviations: FFPE, formalin-fixed paraffin-embedded; HHV-7, human herpesvirus 7; PCR, polymerase chain reaction; CSS, chronic sclerosing sialadenitis.

The respective virus DNA copy numbers in the samples selected for the assay were 16,260 cpm for the HHV-7-positive sample 3,882,433 cpm for the HHV-6B-positive sample, and 2055 cpm for the B19V-positive sample. With RISH we observed HHV-7 U4 signals in the nucleus or cytoplasm of single cells dispersed throughout the tissues, with no specific foci. The signals were located in epithelial cells of striate ducts, lymphocyte infiltrates interspersing the glandular parenchyma, and tissue of the capsule surrounding the gland. Representative signals are shown in Figure 1. The B19V *NS* and the HHV-6 *U38* RISH showed no signals. The qPCR-negative tissue samples remained totally negative for all three viral probes.

## Discussion

In this study, we screened virus DNA from submandibular gland FFPE tissue samples with qPCR to investigate the presence of DNA viruses in the salivary glands of patients with or without CS, with a special emphasis on IgG4-RD and IgG4-overexpressing CS. Furthermore, we performed RISH to visualize the location of the nucleic acids of three viruses, with the highest viral loads by qPCR (HHV-6B and −7, B19V) in the tissue. Viral DNA from 7 viruses (HHV-6B and −7, EBV, B19V, HBoV, VZV, and MCPyV) was present in 48/61 salivary gland samples with co-findings of multiple virus DNAs in 28/61 samples. HSV-1, HSV-2, HHV- 6A, CMV, HHV-8, CuV, BuV, TuV, JCV, BKV, or TSPyV were not found in any of the samples, as could be expected based on current knowledge of the these viruses [20,23,24,30,31,38,39]. There were no significant differences in the virus prevalence or viral DNA copy numbers between the IgG4-positive and -negative groups (Table 1). Additionally, healthy and inflamed tissues often harbored the same viruses (B19V, HHV-6B, and −7, EBV). For HHV-7, both the prevalence and copy numbers were higher in the control group compared to the inflamed tissue.

HHV-6B and HHV-7 DNA were the most frequently detected viruses with a rather equal prevalence within the IgG4-positive (52% and 47%, respectively) and the IgG4-negative samples (47% and 53%, respectively). The control group, which represented healthy salivary gland tissue also showed high prevalences of HHV-6B and −7 DNA (69% and 84%, respectively). These results correlate to the overall reported presence of viruses (DNA) in major salivary gland tissue by PCR: 17–88% for HHV-6B and 42–100% for HHV-7 [18,40–42]. Since HHV-6B and −7 are frequently harbored in healthy salivary glands, an elevated copy number of viral DNA in the inflamed tissue could be associated with disease development, but such a correlation was not observed in this study.

Latent HHV-6B and −7 infections have not been directly associated with disease development in immunocompetent individuals and they are considered rather harmless [20]. Prior studies have investigated the role of HHV-6B and HHV-7 in the pathogenesis of lymphoid lesions and malignancies, such as Hodgkin’s lymphoma, but no association has been established [20,43]. Additionally, HHV-6B and −7 have been reported to enhance the pathological effects and reactivation of other herpesviruses, but the clinical relevance of such a mechanism is not yet fully understood [20,43]. The involvement of these viruses in disease may be more complex, as demonstrated by Jauhiainen et al. [42] where HHV-6B and −7 were present (42% and 58%, respectively) in salivary gland carcinoma ex pleomorphic adenoma but absent in benign pleomorphic adenoma. Our findings are in accordance with earlier studies and further confirm that HHV-6B and −7 persist latently in the salivary glands, even in healthy individuals [18,19]. The present results do not suggest that these viruses would function as an antigen causing chronic inflammation of the submandibular gland or as an antigen triggering the overexpression of IgG4-positive plasma cells in sialadenitis.

A few recent studies have suggested a possible role of EBV in IgG4-RD-related sialadenitis. Furukawa et al. [21] found higher EBV DNA copy numbers in salivary glands with IgG4-RD compared to controls, further the copy numbers correlated with the severity of IgG4-RD. Another study by Takeuchi et al. [22] found only modest EBV positivity (20%) in the samples from patients with IgG4-RD of the submandibular gland. In our study, the prevalence of EBV was higher in the IgG4-positive group (21%) versus the IgG4-negative group (5%), being in line with previous studies [21,22]. Even though the EBV prevalence was higher in IgG4-positive samples, we did not find EBV in any of the three tissue samples from patients with true IgG4-RD. This is somewhat contradictory to the results of Takeuchi et al. and Furukawa et al. [21,22].

EBV initially infects the oropharyngeal epithelium and then persists in memory B cells [44,45]. Even though lymphocyte infiltration is a common feature for non-specific types of sialadenitis [46], Lin et al. [47] found an increased number of memory B cells in the inflammatory infiltrates of submandibular gland samples from patients with IgG4-RD compared to patients with primary Sjögren’s syndrome and healthy controls. Due to its persistence in memory B cells, the presence of EBV DNA in the IgG4-positive samples could hence be in part explained by the dense lymphocyte infiltration and the possible overrepresentation of B lymphocytes in the tissue samples from patients with IgG4-positive sialadenitis, as presented by Lin et al. [47]. Even if none of our three patients with true IgG4-RD were positive for EBV, the finding of EBV DNA in the IgG4-positive sialadenitis could suggest an involvement of such an abnormal B-cell profile. However, further studies are warranted to assess whether EBV has a role in the differentiation of B cells into IgG4-expressing plasma cells or whether the finding of EBV in the tissues of submandibular glands with IgG4-overexpression is merely a result of the unique composition of B-cell subsets present in the inflammatory infiltrates.

The prevalence of B19V in salivary glands has not been extensively studied before, but a recent study by Jauhiainen et al. [42] found B19V DNA by qPCR in salivary gland tumor FFPE samples from three of 25 patients with pleomorphic adenoma and one of 12 patients with carcinoma ex pleomorphic adenoma. In this study, B19V was detected in 20% of the salivary gland samples and seemed to be more frequent in the IgG4-negative samples (26%) than in the IgG4-positive samples (15%), however, with no statistical significance. B19V mainly persists in B lymphocytes and endothelial cells but has only been reported to replicate in erythroid progenitor cells [23,26,48]. Therefore, although B19V DNA was detected in the analyzed salivary gland samples, the virus is most likely only present as transcriptionally inactive genomes inside the infected cells, as shown before in vascular endothelial cells and lymphoid B cells of the intestinal mucosa [48].

We showed that HHV-7 DNA can be visualized *in situ* in major salivary gland FFPE-tissue samples. We did not find previous studies that would have utilized ISH on HHV-7 in salivary glands, thus making this study the first one to visualize HHV-7 distribution in major salivary glands. The HHV-6B or B19V qPCR-positive samples did not show positive dots in RISH, even though high copy numbers of virus DNA (3882433 cpm and 2055 cpm, respectively) were detected in qPCR. The gene transcript used for the HHV-6B probe is only expressed during the replication phase in the acute infection, and since HHV-6B persists latent in the salivary gland tissue, the staining signal might not have been strong enough to be visualized in RNAscope. However, the probes should be able to attach to the genomic DNA strand and thus be visible in RNAscope, even though mRNA is not present [31,48].

This study has some limitations, the first of which is the small cohort size. There were slight differences between the subgroups, which didn’t always reach statistical significance. Second, the presence of viral DNA is not a sign of activity and would require more in-depth investigations, such as mRNA studies, to assess the virus activity. Third, since the DNA in the FFPE samples is more fragmented than in fresh tissue samples and salivary gland tissues with CS might comprise relatively low cellularity due to extensive fibrosis in the tissue [46], the DNA copies might have been undetectable even with the highly sensitive PCR. Furthermore, some regional variation in the virus distribution within the tissue might occur and the punch biopsies represent only a small portion of the salivary gland. Given the retrospective nature of this study, clinical data on comorbidities, including those of the oral cavity, and lifestyle factors such as smoking status, were not available. However, it would be both interesting and important to combine such data to virus findings, as this might shed light on potential associations between oral health events and viruses harbored in the submandibular gland. Even though our results were ultimately inconclusive, a strength of this study is the patient material, which represents a wide range of inflammatory diseases of the submandibular gland, wherein viral etiology has not previously been comprehensively studied. The screening for a versatile group of viruses in this patient material provides useful information on the association of viruses in a variety of inflammatory diseases of the submandibular gland and builds a foundation for further studies.

## Conclusion

DNA of several viruses was present in the salivary glands. The results did, however, not show any substantial difference between the virus profiles of the examined disease entities and healthy tissue. Further, the virus profiles between IgG4-negative and IgG4-positive samples did not differ significantly. Thus, none of the studied viruses seemed to act as an antigen triggering the overexpression of IgG4-positive plasma cells in the submandibular gland. Although the roles of these viruses in the development of CS and IgG4-positive sialadenitis remain inconclusive, this study provides valuable information on the prevalence and persistence of viruses in the submandibular gland and lays the foundations for further studies in larger cohorts.

## Key messages

- The submandibular gland can harbor a variety of different viruses, and the same viruses can be observed in both healthy and inflamed tissue, indicating that many viruses reside latently in the gland rather than acting as active pathogens.
- The viral profiles of IgG4-positive forms of chronic sialadenitis (CS) do not differ from viral profiles observed in IgG4-negative CS, thus viral antigens are not suspected to trigger IgG4-overexpression in CS.
